# Song Practice Promotes Acute Vocal Variability at a Key Stage of Sensorimotor Learning

**DOI:** 10.1371/journal.pone.0008592

**Published:** 2010-01-06

**Authors:** Julie E. Miller, Austin T. Hilliard, Stephanie A. White

**Affiliations:** 1 Department of Physiological Science, University of California Los Angeles, Los Angeles, California, United States of America; 2 Interdepartmental Program in Neuroscience, University of California Los Angeles, Los Angeles, California, United States of America; Max-Planck-Institut für Neurobiologie, Germany

## Abstract

**Background:**

Trial by trial variability during motor learning is a feature encoded by the basal ganglia of both humans and songbirds, and is important for reinforcement of optimal motor patterns, including those that produce speech and birdsong. Given the many parallels between these behaviors, songbirds provide a useful model to investigate neural mechanisms underlying vocal learning. In juvenile and adult male zebra finches, endogenous levels of FoxP2, a molecule critical for language, decrease two hours after morning song onset within area X, part of the basal ganglia-forebrain pathway dedicated to song. In juveniles, experimental ‘knockdown’ of area X FoxP2 results in abnormally variable song in adulthood. These findings motivated our hypothesis that low FoxP2 levels increase vocal variability, enabling vocal motor exploration in normal birds.

**Methodology/Principal Findings:**

After two hours in either singing or non-singing conditions (previously shown to produce differential area X FoxP2 levels), phonological and sequential features of the subsequent songs were compared across conditions in the same bird. In line with our prediction, analysis of songs sung by 75 day (75d) birds revealed that syllable structure was more variable and sequence stereotypy was reduced following two hours of continuous practice compared to these features following two hours of non-singing. Similar trends in song were observed in these birds at 65d, despite higher overall within-condition variability at this age.

**Conclusions/Significance:**

Together with previous work, these findings point to the importance of behaviorally-driven acute periods during song learning that allow for both refinement and reinforcement of motor patterns. Future work is aimed at testing the observation that not only does vocal practice influence expression of molecular networks, but that these networks then influence subsequent variability in these skills.

## Introduction

**Figure 1:**
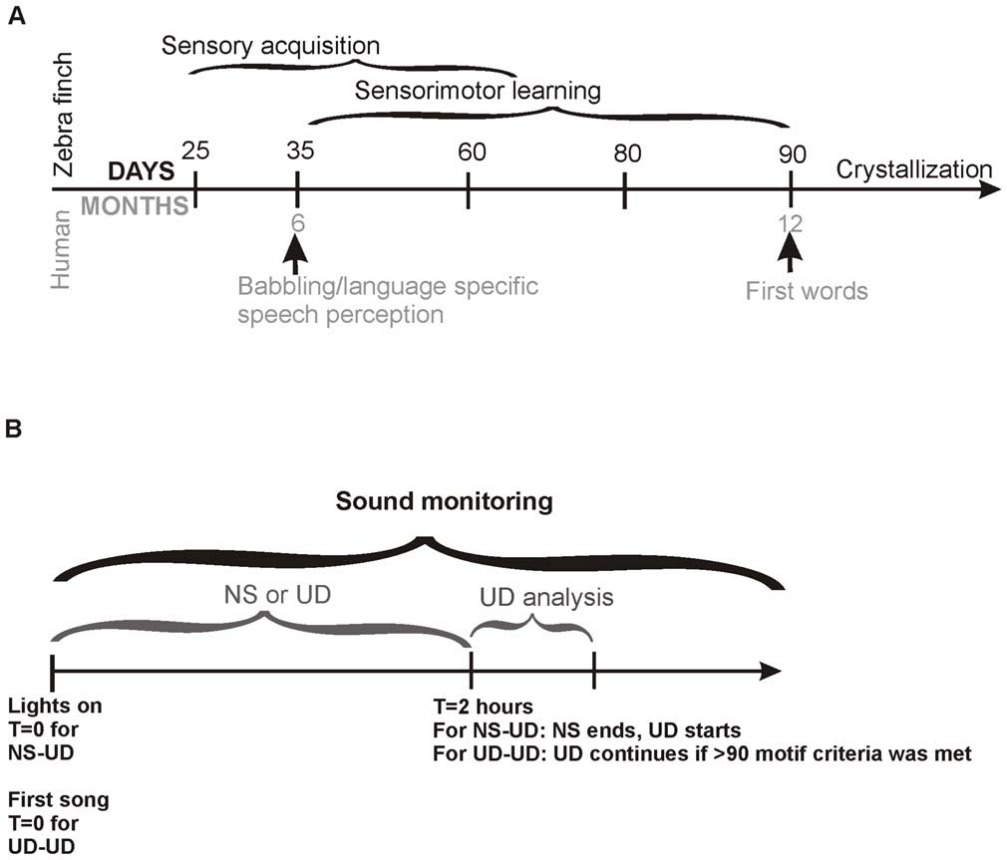
Schematic timelines for vocal learning and for experimental set up. **A**) Relative time frame for zebra finch (top) versus human (bottom) vocal learning (adapted from Doupe & Kuhl, 1999 [4]). Zebra finch song learning occurs over a shorter time scale, requiring only ∼90d for song maturation versus ∼one year for uttering the first word. Sensory acquisition and sensorimotor learning in birdsong correspond to speech perception and babbling, respectively. Experiments in the present study were conducted during late sensorimotor learning at ∼65d and ∼75d. **B**) Following lights-on, individual male zebra finches were assigned to two categories- either 2 hours of non-singing (NS) or 2 hours of undirected singing (UD). On the following day, the bird was assigned to the other condition. At the 2 hour time-point, both groups of birds were allowed to sing uninterrupted undirected song, NS-UD or UD-UD. Analysis was conducted on songs sung during the first 20 motifs or 30 one second clips following the 2 hour time-point. Abbreviations: UD-UD: continuous undirected singing, NS-UD: 2 hours of non-singing followed by undirected singing.

Birdsong and speech share key features [1], [2]. In both, vocal learning is driven by social interactions [3] and occurs during critical developmental periods. In both, learning consists of two often overlapping stages – first, a purely sensory phase and then a sensorimotor phase (Fig. 1A). In the latter, auditory feedback of one's own vocalizations is used to make adaptive modifications which, over time, sculpt them to resemble the vocalizations of conspecifics [4], [5]. At the neural level, this trial and error learning is supported by interactions between the basal ganglia and forebrain. Variability in neural activity, producing motor exploration, enables improved performance and reinforcement of optimum motor patterns [6]–[8]. In contrast to humans, the songbird basal ganglia and forebrain subregions that are dedicated to learned vocalizations are easily identified and well-described. They reside in two interconnected circuits, the posterior vocal motor and the anterior forebrain pathways (AFP). The AFP resembles mammalian cortical-basal ganglia loops for planning and execution of learned complex sequential movements, such as speech [9]–[11].

As zebra finches undergo sensorimotor learning, age-dependent increases in syllable structure are observed between 45–90d [12]. During this time, song phonology (spectral features of syllables; [4], [13], [14]) deteriorates after a night of sleep and recovers following morning singing. Birds with the greatest overall morning deterioration ultimately produce the best copies of their tutors' song [12], highlighting the importance of variability in motor skill learning.

Two nuclei of the AFP, basal ganglia sub-region area X and pallial lateral nucleus of the anterior nidopallium (LMAN), are required for song learning, including processes important for vocal variability underlying motor exploration. Lesions of area X at the onset of sensorimotor learning interfere with song improvement [15] and result in more variable adult songs [16]. Lesions of LMAN early in sensorimotor learning disrupt the motif structure that had emerged prior to the lesion [17]. Changes in song variability that occur over the course of hours to days during normal sensorimotor learning are on a time scale that is consistent with altered transcription of genes. Indeed, undirected singing, a form of vocal practice postulated to lead to song improvement [18], [19], drives changes in the expression levels of certain genes such as Arc, Egr-1, Fos, FoxP2, and Syt IV within song control regions [18]–[29], (Teramitsu et al., companion article).

Among these molecules, the gene that encodes the FOXP2 transcription factor has been a focus of birdsong research because of its direct link to human speech and language [2], [27], [28], [30], [31]. Mutations in this gene produce a disorder with Mendelian inheritance characterized by verbal deficits in phonology and sequential articulation [32]–[34]. FoxP2 expression patterns in human and songbird brain are strikingly similar, including enrichment in the basal ganglia [31]. In zebra finches, RNA-interference mediated knockdown of FoxP2 in area X prior to sensorimotor learning results in abnormal variability in syllable phonological structure and duration in 90d song [35]. Moreover, FoxP2 levels vary naturally as a function of singing: 2 hours of morning song practice down-regulates FoxP2 mRNA and protein in area X [27], [28]. The amount of singing determines the extent of down-regulation, a relationship that is more pronounced in juveniles than adults (Teramitsu et al., companion article). This latter observation is interesting in light of the higher phonological and sequential variability of juvenile song as compared to the highly stereotyped, or crystallized, song of adults. This strong correlation between singing and FoxP2 down-regulation during the developmental time period when song is more variable, and the increased vocal variability of FoxP2 knock-down birds [35] motivated our hypothesis that FoxP2 acts as a ‘plasticity gate’. In this scenario, the low FoxP2 levels during undirected singing (UD, i.e. song practice) promote vocal motor exploration, while higher levels enable reinforcement of optimal motor programs. We thus used these behavioral conditions to determine whether song is indeed more variable following 2 hours of UD singing, than following 2 hours of comparative quiescence. Phonological and sequential features of song were collected in the same birds at 65d and 75d, two ages in late sensorimotor learning. We compared two different methodologies to analyze phonological and syllable sequence variability and validated our findings across a variety of statistical tests.

At 75d, songs were more variable following the condition of vocal practice than following non-singing. Similar consistent trends were observed in these same birds at 65d, but the more variable nature of the developing song precluded detection of significant conditional differences at this age. Accordingly, comparison of songs obtained at 65d and 75d reveal greater stability in these measures at 75d. In a separate group of adult birds, song was even more stereotyped. These findings, together with previous studies, provide insight into the age- and behaviorally-dependent shaping of song variability and motivate additional exploration of behavior – gene interactions.

## Materials and Methods

### Animals

All animal use was in accordance with NIH guidelines for experiments involving vertebrate animals and approved by the University of California at Los Angeles Chancellor's Institutional Animal Care & Use Committee. Juvenile ∼62 days of age (62d) or adult (125–160d) male zebra finches were moved from our breeding colony to individual sound attenuation chambers (Acoustic Systems; Austin, TX) under a 12∶12 hour light/dark cycle. Birds were left undisturbed for 2–3 days prior to the behavioral experiments to enable acclimation to the new environment.

### Song Recording

Sounds were recorded using Shure SM57 microphones and digitized using a PreSonus Firepod (44.1 kHz sampling rate, 24 bit depth). Recordings were acquired and analyzed using Sound Analysis Pro 2.091 software with pre-set parameters for capturing zebra finch song (SAP; [36]). Songs were pre-screened from males housed alone, singing in a solo context, also known as ‘undirected song’ (UD; [37]). Songs were examined for the presence of motifs, a kernel of acoustic structure defined by a repeated sequence of syllables. Birds whose songs lacked identifiable motifs were excluded from the study.

UD song was recorded and analyzed from the same bird at two stages late in sensorimotor learning (Fig. 1 B), referred to henceforth as 65d (range: 64d–68d) and 75d (range: 74–76d). Birds remained singly housed during the ∼8–10 days between the two ages. Songs were also recorded from a separate group of singly housed adult males (see below). We previously found that these housing conditions did not alter stress levels as measured by serum corticosterone values in different contexts including in the presence of the investigator [28].

### Behavior

Our prior work showed that 2 hours of UD singing lowers levels of FoxP2 mRNA and protein within area X of male zebra finches [27], [28], (Teramitsu et al., companion article). A minimum criterion of 90 motifs sung within 2 hours was sufficient to induce this down-regulation. By contrast, FoxP2 levels remain high following 2 hours of non-singing (NS). For the current study, using these criteria, we collected and compared song immediately following 2 hours of UD song or non-singing conditions in birds at 65d and 75d as well as in a separate group of adult birds. Necessarily, FoxP2 levels were not re-measured here as obtaining them via in situ hybridization analyses on brain tissue would, by definition, preclude subsequent behavioral analyses, which was the focus of the present study. Experiments were conducted in the morning from the time of lights-on (Fig. 1 B), following general methods in Miller et al. (2008) [28]. Each bird underwent the following conditions on adjacent days (Fig. 1 B):

1. Non-singing then undirected singing (NS-UD): For the first 2 hours following lights-on, the door to the sound chamber was propped open and birds were monitored by the presence of the investigator nearby, and distracted if they attempted to sing. If distraction was ineffective such that birds sang >10 motifs during the experiment, they were excluded from the study. After 2 hours, the chamber door was closed and the bird left undisturbed. Songs sung immediately after this 2 hour timepoint (see below) were used for behavioral comparisons. The time of the first subsequent UD motif was usually shortly after door closure (for example, 75d range: 1–25 min, mean = 7.5 min, n = 11).
2. Undirected singing throughout (UD-UD): UD song was continuously recorded from the time of lights-on and throughout the morning. The time of the first motif was usually shortly after lights-on (75d range: 1–14 min, mean = 5 min, n = 11). Two hours thereafter, song was immediately collected for behavioral comparisons.

At all ages, half of the birds in the group were in the NS-UD condition on Day 1 and the UD-UD condition on Day 2. This order was reversed for the other half. Counterbalancing was done to ensure that any changes in song structure were due to a conditional or age effect and not due to the chronological order of recordings. Ten birds were successfully recorded at 65d and 11 birds at 75d with 9 of these birds recorded at both ages. One bird was successfully recorded at 65d but not at 75d because of repeated singing during the non-singing period. Two birds recorded at 75d were not recorded at 65d due to technical problems. Methods for juvenile song analysis are detailed in the next section, followed by adult song analysis.

### Song Analysis: Juveniles

#### Motif based vs. non-motif based analyses

In this study, we present two separate methods for analyzing the same behavioral data obtained within the first 30 minutes following an initial 2 hours of either non-singing (NS-UD), or undirected singing (UD-UD). One method relied on investigator-defined segmentation of motif structure, while the second was independent of such judgments. In the first method, referred to as ‘motif-based’, we quantified phonological and sequence variability within the context of the motif, considered to be the basic analytical unit of song encoded by specific patterns of neuronal firing [38]. By focusing on the motif, we sacrificed some objectivity (as the investigator assessed what constituted a motif) and statistical rigor (compared to our other analyses; see below), although perhaps ultimately providing more ethologically relevant results. For each bird, phonological and sequential variability in 20 segmented motifs immediately following the two hour timepoint were analyzed using asymmetric comparisons which enable comparison of the most similar sound elements in the two motifs, independent of their position (Song Analysis Pro Manual; [35]). At the level of syllables, scores for similarity, accuracy, and individual acoustic features were obtained for 2–3 individual syllables within these motifs from 25 consecutive renditions (see sections on ‘individual syllables’ below) using symmetric comparisons. This latter analysis enables comparison of a single frame of one sound element or syllable to another (Song Analysis Pro Manual).

In the second method, phonological variability was assessed using 30 one second song clips (‘clip-based’), while sequence variability was assessed using the first 300 syllables (‘string-based’), similar to the method used by Haesler et al. [35]. Since the motifs of some birds were less than one second long, the scores resulting from the clip-based analysis often used twice as much acoustic data as the scores from the motif-based analysis. Also, since a given bird may have multiple versions of his motif, each with different lengths, taking one second clips controlled for the length of the song samples analyzed in SAP. We present the results of both methods and discuss possible reasons for any differences (see Appendix S1).

### Phonological Analysis: Acoustic Features of Individual Syllables

In addition to comparing entire motifs, motifs were examined for selection of 2–3 individual syllables for more detailed comparisons. Local similarity and accuracy scores for 25 examples of each of these syllables were computed using symmetric pair-wise comparisons in SAP [35]. Syllables were selected based upon their being present in multiple motifs (at least 25), being easily distinguishable from other syllables in the motif, and for their acoustic properties. For the latter, syllables that had flat harmonics (little or no frequency modulation, high pitch goodness and low Wiener entropy) as well as those that had little or no harmonic structure (high Wiener entropy) were selected in order to obtain a diverse representation of the motif data and reliable calculations [12], [35], [39], [40]. A given bird's selected syllables were analyzed in each condition, and at each age (although syllables were not always present at both ages, as noted in the text and statistics). For 2 birds, only 1–2 syllables fit these criteria. In total, 25 syllables from 10 birds were analyzed at 65d while 30 syllables from 11 birds were analyzed at 75d. For the 65d versus 75d age comparisons, 22 syllables common to both ages were compared.

Mean syllable scores were obtained in two different ways– means were either computed for each bird (i.e. mean of 1–3 syllables) and then collapsed across birds or a single mean was derived from all syllables independent of the birds . The rationale for selecting either method was as follows. A bootstrap 1-way ANOVA determined that the bird, not the syllable, was the independent factor when assessing similarity and accuracy (p<0.05 for between-bird differences). Thus, for syllable similarity, accuracy, and identity (product of similarity×accuracy/factor of 100) scores, the mean values and statistical tests reported in the tables are based on average scores per bird (n = 10 at 65d, and n = 11 at 75d). The case was different for the CV scores such that syllables were independent of the bird. Given the lack of significant conditional effect for the syllable similarity and accuracy scores at 65d (see Results, below), CV scores for individual acoustic features were only conducted on data obtained at 75d.

Wav files representing 25 renditions of each of the selected syllables at 75d in both conditions were manually segmented using amplitude and entropy thresholds in the sound analysis window of the similarity features tab of SAP. These features are: duration, amplitude, frequency modulation (FM), pitch, Wiener entropy, mean frequency, and pitch goodness. The coefficient of variation (CV, standard deviation/mean of 25 renditions) is reported (n = 30). The mean, standard deviation (SD), standard error of the mean (SE), and CV from 25 renditions were calculated for each syllable and then averaged for all 30 syllables, since the above mentioned 1-way bootstrap ANOVA indicated that the syllable was the independent variable for this analysis (p>0.05).

#### Phonology: motif-based

The first 20 motifs sung after 2 hours of non-singing (NS-UD) or UD singing (UD-UD) were selected by visual inspection of spectrograms in Audacity (version 1.3; http://audacity.sourceforge.net/). Extraneous noise (e.g. from chamber fans) was filtered out using the built-in high-pass filter. Two measures of phonological (spectral features of song; [13], [4], [14]) variability were obtained from SAP: similarity and accuracy scores, computed using asymmetric comparisons of time course. SAP calculates the mean absolute deviation of pitch, frequency modulation (FM), Wiener entropy, and pitch goodness, and computes the Euclidean distance between these measures between samples. A short Euclidean distance indicates that there is little variation between samples, producing a high similarity score. The similarity score is computed from segments of 70ms windows, whereas the accuracy score is the mean of the local similarity scores derived from smaller 9ms windows. In SAP, syllable segmentation parameters were optimized for each set of 20 motifs. Then, for each bird in each condition (NS-UD or UD-UD) at each age (65d or 75d), 20 motifs were run against themselves excluding comparison to self, using SAP's batch module (m×n comparisons) to generate self-accuracy and self-similarity scores. This produced a list of scores (20×20)−20 self-tests = 380 scores for motifs from which the mean and SE were obtained, indicative of the bird's acoustic variability for that condition/age.

#### Phonology: clip-based

Songs were divided into 30 one second clips (adjusting for syllable boundaries as needed, never more than+/−0.1 sec) and analyzed in SAP to quantify phonological variability. We used the 75d NS-UD data from each bird to set syllable segmentation parameters in SAP, since the motif analysis indicated that singing in this condition was the least acoustically and sequentially variable. These parameters were then held constant for analysis of that bird's other three datasets (75d UD-UD, 65d NS-UD, 65d UD-UD) to account for possible subtle changes in syllable formation. The choice of 30 samples was based on empirical discovery of the minimum number of one second samples needed to provide a stable average score, as follows. We gradually increased the number of samples compared from each condition (starting with 10 samples and incrementing by 5 each time) until the mean similarity and accuracy scores no longer changed. As in the motif analysis, each one second clip was compared to all other samples collected in that condition, ((30×30)−30 self-tests = 870 scores for one second clips) from which the average was taken to produce scores of self-similarity and self-accuracy for a given age and condition.

### Sequence Analysis: Overview

Quantification of sequence variability was performed by first estimating the transition probability distributions of individual syllables, then calculating the scaled entropy of the distribution for each syllable, and finally averaging across syllables to obtain a score of motif entropy [16], [35]. An entropy score of 0 indicates that the motif syllable sequence is fixed, and as entropy approaches 1, the sequence becomes more variable. Motif stereotypy is defined as “1 – entropy”, thus a stereotypy score of 1 indicates fixed syllable order.

The transition probability is a ratio representing the number of times that each leading syllable transitions to some following syllable (including to itself) over many renditions, divided by the total number of occurrences of the leading syllable. Thus, the transition probability of syllable A to B is defined as
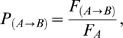
where *F_(A→B)_* is the number of times leading syllable A is followed by syllable B, and 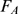 is the total number of syllable A in the data. All transition probabilities between syllables can be conceptualized as a Markov chain [41]. Transition entropy for a given syllable is defined as
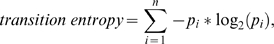
where *n* is the total number of unique syllables, and *p_i_* is the probability that a given leading syllable is followed by syllable *i*. Since the sum is over all unique syllables, the number of unique syllables sung by a given bird can greatly affect the raw entropy score. To account for this, we normalized the transition entropy of each syllable to the maximum possible entropy of that bird's motif, then averaged the normalized syllable entropy scores to obtain the motif entropy. Syllable entropy is maximized when it is equally likely for that syllable to transition to any of the other syllables in the motif. For example, if a bird has 5 unique syllables, the entropy of a given syllable is maximal if it is equally likely (20% probability) for that syllable to be followed by itself or any of the other syllables. Thus,
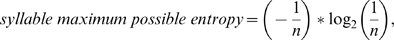
where *n* is the number of unique syllables in the bird's motif. It follows that
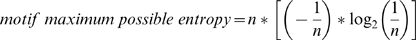



Since motif entropy is an average of normalized syllable entropy scores, the entropy of each syllable contributes equally to the motif score. This may be problematic if a certain syllable only appears rarely but has a very high or very low entropy, since this skews the motif entropy towards the entropy of the rarely occurring syllable, especially if the bird has only a few unique syllables to begin with. We addressed this potential skewing in the string-based analysis, described below.

During the 300 syllable ‘string-based’ analysis, we set syllable segmentation parameters in SAP based on the bird's 75d NS-UD data, and then allowed SAP to automatically define syllables in all of that bird's subsequent datasets. Setting these parameters once using the most precise version of the song available allowed for a more objective definition in the other data sets. For some birds, syllables that were frequent at 65d, were rare at 75d due to their having merged into a single new syllable over the ten day interval. We also observed single syllables in the 65d data that were split into separate syllables at 75d. Most of the time, the 65d syllables did not disappear altogether but simply appeared much less frequently in the 75d data. Usually, these infrequently occurring syllables had entropy scores approaching 1 or 0, likely due to the small sampling rate that strongly drove the motif entropy score.

One way to deal with this skewing would have been to exclude infrequently occurring syllables from the analysis, but it is not clear what an appropriate occurrence threshold for excluding infrequent syllables would be. Because of that uncertainty, and more importantly, in order to capture the full complexity of the song, we chose not to do this. Instead, we created a weighted entropy score for the string-based analysis. The weighted entropy was obtained by calculating the normalized syllable entropy as described above, then weighting syllable entropy by the ratio of how frequently that syllable was sung, relative to the most frequent syllable. Thus,
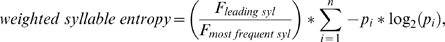
where 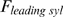 = the number of times the syllable in question was sung and 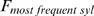 = the number of times the most frequently occurring syllable was sung. The normalized, weighted syllable entropies were then averaged to ensure that infrequently occurring syllables did not over-influence the score. Both the standard and weighted scores are reported.

#### Sequence analysis: motif-based

Individual syllables were defined by the investigator while viewing song spectrograms in Audacity. Each unique syllable was designated a letter name, then letter names were translated into numbers for analysis in MATLAB, where A = 1, B = 2, C = 3, etc.. Plain text files were created with each line representing a motif as a string of numbers. Each bird had one 20 line text file per dataset available, representing the first 20 motifs sung immediately following 2 hours of non-singing (NS-UD) or undirected singing (UD-UD) at each age, if available. MATLAB functions (see Appendix S1) were written to automate calculation of syllable and motif entropy and stereotypy from these text files. To capture as much information as possible about syllable sequence, transitions to the end of a motif were included in the calculations for this analysis. We calculated motif entropy and stereotypy, as well as the percent change from the NS-UD to the UD-UD condition.
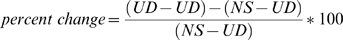
The percent changes are presented as histograms in order to illustrate any positive or negative shifts. Stereotypy scores presented in the tables were multiplied by 100 to facilitate comparison to similarity and accuracy measures.

#### Sequence analysis: string-based

In this analysis, we calculated syllable transition probabilities from the first 300 SAP defined syllables that were sung immediately following 2 hours. This usually corresponded to slightly more than one minute of continuous song. Individual syllables were segmented automatically in SAP using the pre-set parameters [36], then labeled with numbers in Audacity, and finally exported as a string of 300 numbers into MATLAB for analysis. Using variations of the same MATLAB code used in the motif analysis, we calculated unweighted and weighted syllable and motif entropy and stereotypy scores. In this analysis, we excluded transitions to the end of a motif in the calculations since our data collection procedure ignored motif structure to begin with, and no information about motif termination was included in the data presented to MATLAB. That is, the input data was only one long string of syllables, instead of 20 separate lines of syllables. Sequence stereotypy was defined as above.

### Adult Analysis

As in juveniles, two hours of morning UD singing decreases area X FoxP2 levels in adults relative to levels following two hours of non-singing [27], [28]. These adult data formed the initial impetus for examining vocal changes following UD singing or non-singing. Thus, we began this work by examining any behaviorally driven changes in six adults (125–160d) using similar methods as described above. Motif similarity, accuracy, and stereotypy scores were obtained from 20 motifs per bird/per NS-UD or UD-UD condition immediately following the 2 hour time-point. However, we failed to detect any significant conditional differences in adults (see Results and Discussion). Subsequently, we discovered a more robust correlation between the amount of song and FoxP2 levels in area X at 75d (which is only a trend in the adult data; Teramitsu et al., companion article). Retrospectively, this motivated our work in the juveniles at two ages for each bird during late sensorimotor learning.

### Statistics

In traditional statistical methods, a test statistic such as Student's t or Fisher's F is compared to a mathematically derived continuous probability distribution of that statistic under the null hypothesis. In resampling statistics (also known as ‘bootstrapping’ and referred to henceforth) the appropriate statistic is defined by the experimenter and then compared to a distribution of that statistic under the null hypothesis which is generated via randomly permuting the data. In this way, bootstrap tests avoid the need for any theoretically based assumptions as to the form of the real data to assure validity of the test, as in traditional parametric statistics. We performed bootstrap tests using custom MATLAB functions (written by ATH; see Appendix S1), using the mean of paired differences as the test statistic. These tests are comparable to the paired t-test, and are valid in cases that violate the assumptions of standard statistical tests [42]. We began by calculating the group mean of the individual birds' conditional (NS-UD vs. UD-UD) differences. This mean was our test statistic, M. Then, we randomly sampled *n* times from a vector containing 1 and −1, where *n* was the number of birds. The *n* element long vector of 1's and −1's was multiplied by the vector containing the actual differences, effectively randomizing the direction of the conditional differences. Then, we took the mean of this randomized data and repeated the randomization process 10,000 times, keeping track of the mean each time. These means formed the distribution of M under the null hypothesis, reflecting the values of M we could have expected if the direction of the individual conditional differences was random, and was not an effect of the experimental paradigm.

Finally, the number of Ms in the null distribution outside the critical values (actual M and its reflection across the mean of the null distribution) divided by 10,000, was the likelihood that we could have observed such a difference if there were no real conditional effect. This likelihood is the p-value. In contrast to a traditional t-test that has a critical t value indicating statistical significance at some alpha level, the critical M values change depending on the null distribution generated in each test, although alpha (0.05) does not. The same basic procedure, with a different test statistic, was used in the bootstrap 1-way ANOVAs to assess the independent variable, bird or syllable, in the syllable analysis as described above. The test statistic in this case was the ratio of between-group over within-group variability, computed not as sums of squares as in a traditional F-test, but as sums of absolute values of the distances from the grand/group means.

Since we could not confirm a normal distribution for many of our datasets, thus not satisfying a major assumption of traditional t-tests, we performed non-parametric 2-tailed paired bootstrap tests on all data (see Tables 1, 2, 3, 4, 5, S1, S2, S3) along with 1-way bootstrap ANOVAs, as described above.

**Table 1:** Scores for conditional comparisons.

Condition Comparison	p-value					p-value				
	75d NS-UD	SE	75d UD-UD	SE	75d NS-UD vs. UD-UD	65d NS-UD	SE	65d UD-UD	SE	65d NS-UD vs. UD-UD
Motif Similarity	85.74	1.25	82.69	1.52	**<0.0005**	83.76	1.36	83.53	1.18	0.878
Motif Accuracy	83.10	0.71	81.75	0.60	**0.001**	79.92	0.70	79.97	0.78	0.932
Motif Entropy	0.29	0.03	0.35	0.05	**0.032**	0.42	0.06	0.44	0.07	0.603
Motif Stereotypy	70.69	3.34	64.95	4.59	**0.036**	57.78	5.84	55.56	6.88	0.596
Clip Similarity	82.45	1.17	80.25	1.24	0.064	84.51	1.04	82.44	1.44	**0.018**
Clip Accuracy	80.20	0.42	79.39	0.39	**0.020**	78.16	0.71	77.89	0.78	0.689
String Entropy	0.28	0.03	0.34	0.04	**0.003**	0.34	0.04	0.38	0.04	0.183
String Stereotypy	72.45	3.49	66.10	4.22	**0.003**	65.97	3.96	62.23	3.97	0.174
Syllable Similarity	96.95	0.38	95.88	0.62	**0.004**	95.31	0.81	94.84	0.71	0.408
Syllable Accuracy	92.79	0.37	91.92	0.47	**<0.001**	91.71	0.63	91.38	0.57	0.543
Syllable Identity	89.97	0.70	88.16	1.00	**<0.0001**	87.46	1.32	86.70	1.16	0.467

Mean scores with standard error (SE) and exact p-values for 2-tailed paired bootstrap tests (significant p-values in bold face type) are shown for phonological and sequence comparisons between conditions generated at 75d (n = 11, left columns) and 65d (n = 10, right columns). Results are first reported for motif-, clip-, and string-based analyses, followed by syllable scores. For the latter, the investigator selected 25 consecutive renditions of the same syllable, computed an average of ∼3 syllables per bird and obtained the mean from 10 birds at 65d and 11 birds at 75d.

**Table 2:** Coefficient of variation in individual acoustic features at 75d.

CV, Individual Syllables (n = 30)	NS-UD	SE	UD-UD	SE	p-value
Pitch*	0.074	0.007	0.084	0.008	**0.022**
Pitch Goodness*	0.101	0.006	0.123	0.100	**0.004**
Wiener Entropy*	0.085	0.005	0.098	0.008	**0.021**
Syllable Amplitude*	0.034	0.003	0.043	0.003	**0.005**
Syllable Duration	0.064	0.007	0.062	0.008	0.638
Frequency Modulation (FM)	0.118	0.013	0.125	0.011	0.379
Mean Frequency	0.064	0.006	0.065	0.006	0.984

The coefficient of variation (CV, standard deviation/mean) with SE is reported for all features obtained from 25 syllable renditions per bird in the NS-UD (left columns) or the UD-UD (right columns) condition. Asterisks and bold face type denote significance by 2-tailed paired bootstrap test.

**Table 3:** Power analysis of 65d and 75d data.

Motif analysis		Power	Clip analysis		Power
75d- NS vs. UD	similarity	76.5%	75d- NS vs. UD	similarity	44.5%
	accuracy	96.7%		accuracy	63.2%
	stereotypy	52.0%		stereotypy	76.3%
	weighted stereotypy	52.4%		weighted stereotypy	51.7%
65d- NS vs. UD	similarity	7.0%	65d- NS vs. UD	similarity	62.2%
	accuracy	4.7%		accuracy	8.2%
	stereotypy	11.2%		stereotypy	26.4%
	weighted stereotypy	10.5%		weighted stereotypy	8.8%
NS- 65d vs. 75d	similarity	11.4%	NS- 65d vs. 75d	similarity	49.4%
	accuracy	78.3%		accuracy	65.5%
	stereotypy	72.8%		stereotypy	65.7%
	weighted stereotypy	56.5%		weighted stereotypy	32.4%
UD- 65d vs. 75d	similarity	15.2%	UD- 65d vs. 75d	similarity	18.5%
	accuracy	63.6%		accuracy	49.5%
	stereotypy	42.4%		stereotypy	42.7%
	weighted stereotypy	27.2%		weighted stereotypy	7.3%

Results for the power analysis are shown for the 65d and 75d data. Higher power is seen in the ability to detect conditional differences between NS-UD and UD-UD in the 75d data relative to the 65d data which exhibits high variability within each condition. A comparison of the 65d vs. 75d data within a condition reveals higher power to detect age differences in the NS-UD condition.

**Table 4:** Scores for age comparison.

Age Comparison: 75d vs. 65d	75d		65d		p-value	75d		65d		p-value
	NS-UD	SE	NS-UD	SE	75d vs. 65d	UD-UD	SE	UD-UD	SE	75d vs. 65d
Motif Similarity	85.15	1.46	84.05	1.49	0.514	82.17	1.82	83.69	1.37	0.334
Motif Accuracy	82.66	0.69	80.11	0.75	**0.002**	81.46	0.66	79.89	0.87	**0.016**
Motif Entropy	0.27	0.03	0.44	0.06	**0.001**	0.34	0.05	0.45	0.08	0.101
Motif Stereotypy	72.65	3.32	56.30	6.31	**0.008**	66.32	5.46	54.84	7.64	0.099
Clip Similarity	82.28	1.24	84.73	1.14	0.052	80.53	1.48	82.40	1.61	0.298
Clip Accuracy	80.04	0.51	78.17	0.79	**0.008**	79.44	0.48	77.81	0.87	0.052
String Entropy	0.27	0.04	0.35	0.04	**0.009**	0.33	0.05	0.39	0.04	0.073
String Stereotypy	73.35	4.13	64.87	4.26	**0.008**	67.21	5.10	60.78	4.13	0.065
Syllable Similarity	96.78	0.47	95.24	0.90	0.106	95.55	0.77	94.71	0.78	0.321
Syllable Accuracy	92.57	0.48	91.61	0.70	0.177	91.74	0.62	91.17	0.59	0.249
Syllable Identity	89.96	0.77	88.06	1.33	0.135	88.19	1.10	86.94	0.95	0.293

Within each condition, mean scores with SE and exact p-values for 2-tailed paired bootstrap tests (significant p-values in bold face type) are shown for phonological and sequence comparisons between ages for 9 birds. Age comparisons for the NS-UD condition are on the left half of the table, while those for the UD-UD condition are shown on the right. Results (top to bottom) are for motif-, clip-, and string-based analyses, followed by syllable scores.

**Table 5:** Adult data and power analysis.

Motif analysis	NS-UD	SE	UD-UD	SE	p-value, NS-UD vs. UD-UD	Power
similarity	92.10	1.13	93.27	1.59	0.136	33.0%
accuracy	86.04	1.76	86.69	1.57	0.534	18.0%
entropy	0.11	0.02	0.11	0.02	0.950	5.6%
stereotypy	0.89	0.02	0.89	0.02	0.951	4.7%

Within each condition, mean scores with SE and exact p-values for 2-tailed paired bootstrap tests are shown for phonological and sequence comparisons in adult data between NS-UD and UD-UD conditions. All measures showed low power reflecting a lack of conditional differences.

Figures and tables were created in Microsoft Excel, JMP (Cary, NC), and Origin (Northampton, MA). To enable comparison of traditional parametric and nonparametric statistical approaches with nonparametric bootstrap statistics, we report the results of all tests in Tables S1, S2, S3. For ease-of-reading, p-values for only the bootstrap test are reported in the text. All results reported as statistically significant in the bootstrap tests were so at the p<0.05 level also by 2-tailed Wilcoxon signed rank test, which further validated our findings. A power analysis was performed on the 65d, 75d and adult data using MATLAB code (ATH) to determine the power to detect conditional or age differences (Tables 3, 5). The power value is impacted by 3 major factors: the variability within-group, the difference between groups, and the number of birds, *n*. Power is also affected by other parts of the experimental design, such as the significance threshold alpha, which we set at the conventional 0.05 level.

## Results

Songs that were sung by males immediately following a 2 hour period of UD singing (UD-UD) were compared with those sung following 2 hours of non-singing (NS-UD) at two ages in late sensorimotor learning (65d and 75d). In line with our prediction, phonological and sequential measures of song variability were higher in the UD-UD condition at 75d. At 65d, similar trends were evident but detection of significant conditional differences was overshadowed by higher within-condition variability. Song features in both conditions increased in stability from 65d to 75d, reflecting age-specific coarse- and fine-tuning of these processes. In contrast, a separate group of adult birds showed highly stereotyped song in both conditions.

### Syllables Become More Variable after Continuous Vocal Practice at 75d

**Figure 2:**
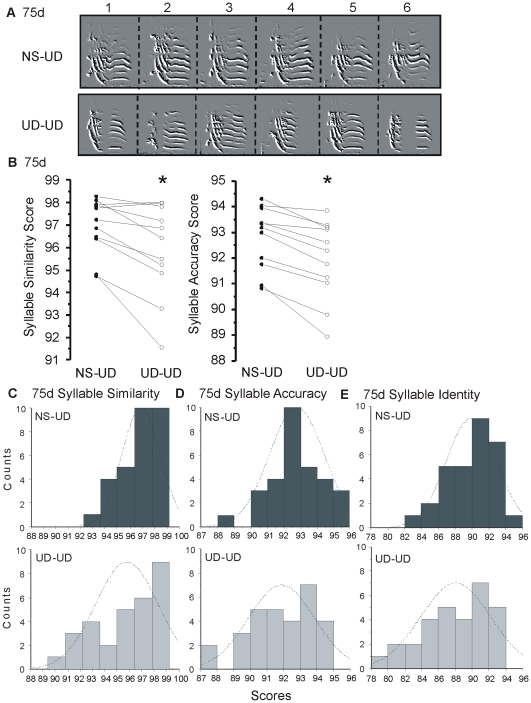
Continuous song practice increases syllable variability at 75d. **A**) Examples from one bird at 75d show spectral derivatives of syllables. Six renditions (1–6) of the same syllable are illustrated with time on the x-axis, and frequency on the y axis. In the UD-UD condition, the syllable is composed of two disjointed notes in 1,2,6, but as one continuous note in 3–5 and in all of the NS-UD renditions. **B**) Paired data scores for the NS-UD (filled circles) and UD-UD (open circles) conditions for each bird are represented by connected lines. The UD-UD condition had lower (*) mean syllable similarity and accuracy scores (p<0.005, p<0.001, 2-tailed paired bootstrap). **C–E**) Histograms for all 30 syllables representing 11 birds are shown with similarity, accuracy, and identity scores on the x-axis and counts (frequency of occurrence) on the y-axis. The dashed line shows what a fitted curve through a normal distribution would be and is provided for comparison to the actual data. The UD-UD scores (light grey bars) are more broadly distributed and shifted towards lower scores than are the NS-UD scores (dark grey bars) (2-tailed paired bootstrap for similarity, accuracy, and identity, p<0.0001).

At 75d, with continuous vocal practice (UD-UD), phonological features of the notes within a given syllable often appeared altered relative to one another (see Fig. 2 A exemplar); a phenomenon that seemed rarer in the NS-UD condition. To quantify this, we examined the variability of single syllables against themselves and compared this feature across conditions. As predicted, phonological features of syllables exhibited greater variability in the UD-UD condition relative to the NS-UD condition (n = 11 birds, similarity, p<0.005; accuracy, p<0.001; identity p<0.0001; Fig. 2 B, Table 1 bottom). Figure 2 (C–E) shows the distribution of scores for every syllable that was analyzed in these 11 birds. In the UD-UD condition, the distribution of scores was broader and shifted to the left relative to the NS-UD condition (n = 30 syllables, p<0.0001).

**Figure 3:**
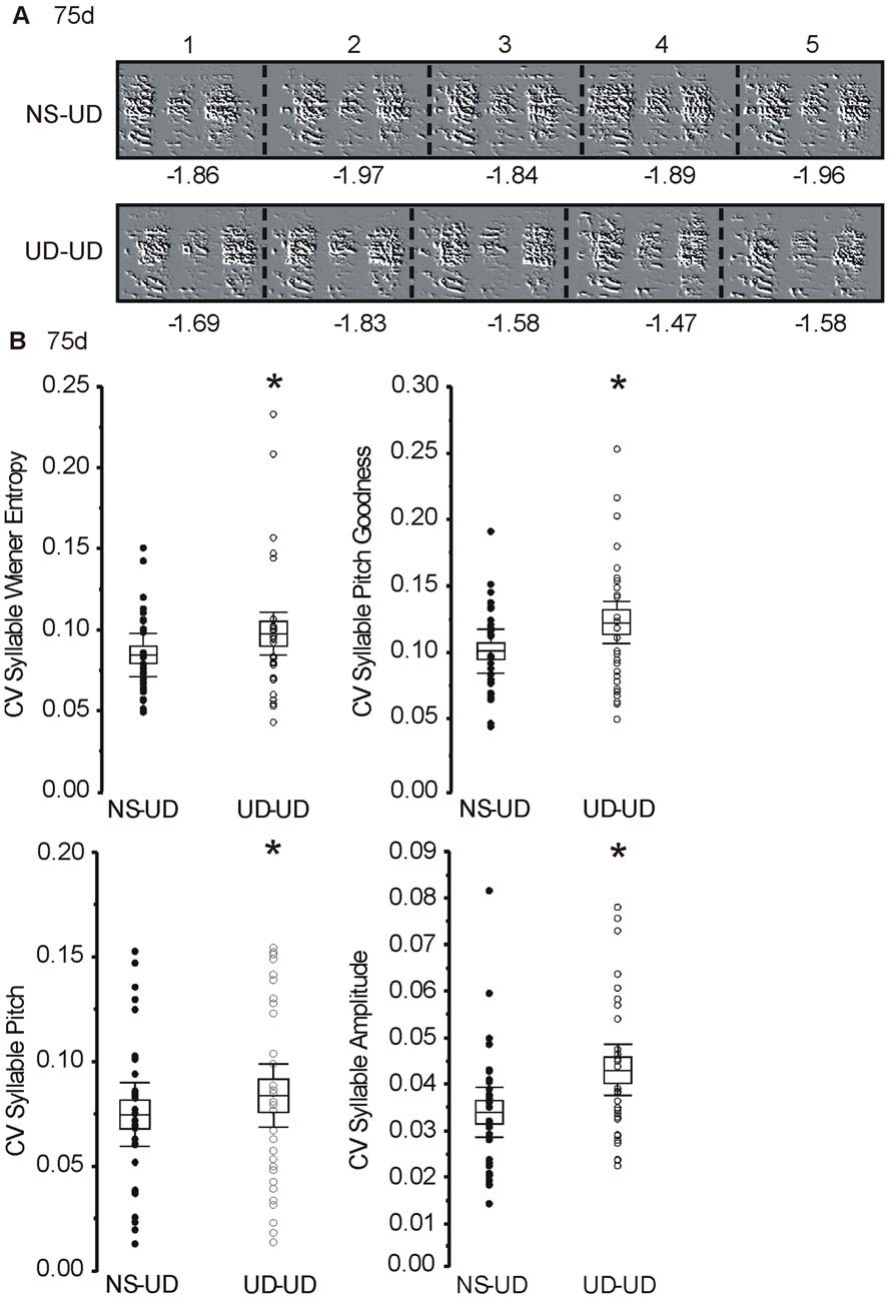
Continuous song practice increases variability in several acoustic features of syllables at 75d. **A**) Examples from another bird at 75d reveal more variation in Wiener entropy from rendition to rendition in the UD-UD condition. Five consecutive renditions of the same syllable are shown in both panels. Each syllable consists of 3 notes which alter their spectral appearance from rendition to rendition. Wiener entropy is more variable, as reflected in the higher CV in the UD-UD condition than in the NS-UD condition. Numbers beneath are entropy scores, with more negative values indicative of less entropy (more spectral order). Zero is maximum entropy and negative infinity is maximum order. **B**) Box plots show the mean scores (middle of the box), standard error (top and bottom of the box), and upper and lower 95% confidence intervals (whiskers). Data scores for the NS-UD (filled circles) and UD-UD (open circles) conditions for ∼3 syllables from each bird are represented by individual points. Mean CV scores were obtained from 25 renditions of the same syllable. The UD-UD condition had higher CV values (*; 2-tailed paired bootstrap) for amplitude (p<0.005), pitch (p<0.05), pitch goodness (p<0.005), and Wiener entropy (p<0.05). Removal of points that are greater than two standard deviations above the mean does not remove the significance.

We next examined the mean coefficient of variance (CV) in these 30 syllables. As predicted, the CV was higher in the UD-UD condition for individual syllable features (pitch, p<0.05; pitch goodness, p<0.005; Wiener entropy, p<0.05; syllable amplitude, p<0.005; Fig. 3 B, Table 2). Figure 3A shows a representative example of the higher Weiner entropy observed in the UD-UD condition. No differences were observed in syllable duration, FM, or mean frequency (p>0.05; Figure S1). Although the increased CV of pitch observed in the UD-UD condition supported our hypothesis, we had some concern about making pitch measurements on syllables with high frequency modulation. We thus planned to separately analyze a subset of syllables that had flat harmonic stacks. However, only 9 such syllables were reliably identified, and statistical tests revealed that this sample size only had 12% power. We were thus unable to make meaningful comparisons on this subset of data.

### Continuous Vocal Practice Increases Song Variability at 75d

**Figure 4:**
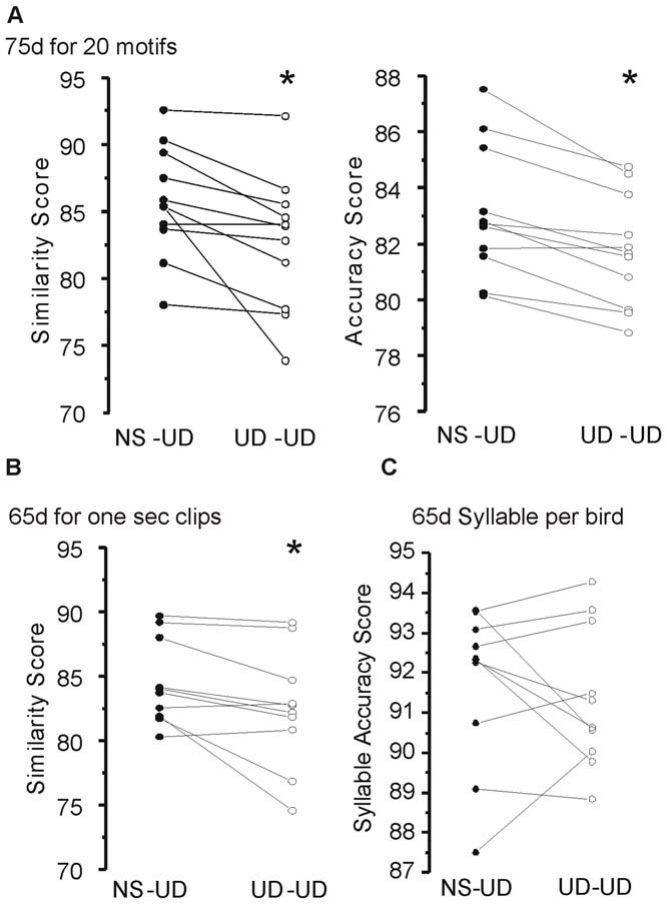
Additional motif- and clip-based analyses confirm that continuous song practice increases variability. Paired data scores for the NS-UD (filled circles) and UD-UD (open circles) conditions for each bird are represented by connected lines. **A**) Motif similarity (left) and accuracy (right) scores for 75d birds (n = 11) are shown by condition. The UD-UD condition had lower (*, 2-tailed paired bootstrap) similarity (p<0.0005) and accuracy scores (p<0.001) compared to the NS-UD condition. **B**) Similarity scores were also lower at 65d in the UD-UD condition using the clip-based analysis (p<0.05). **C**) Individual points represent the mean syllable accuracy score per bird (p>0.05).

Akin to the individual syllable analysis at 75d, the motif- and clip-based metrices also revealed greater variability in the UD-UD condition. These analyses compared a series of song syllables, including syllables used for the analysis of individual acoustic features detailed above. As described, motif similarity and accuracy scores are derived from calculations of individual acoustic features in SAP, including pitch, FM, Wiener entropy, and pitch goodness. 75d birds in the UD-UD condition, representing uninterrupted vocal practice, had lower mean similarity and accuracy scores, indicative of higher phonological variability, than in the NS-UD condition (n = 11, similarity, p<0.001; accuracy, p<0.001; Fig. 4 A; Table 1 top). The clip-based analysis corroborated the motif-based findings, revealing greater variability in the UD-UD condition for accuracy (p<0.05) and a trend for a lower similarity score in the UD-UD condition (p = 0.064; Table 1 middle).

### Greater Sequence Variability after Continuous Vocal Practice at 75d

We utilized similar entropy-based methods as in Haesler et al [35] to measure sequential variability, investigating the first 300 syllables (string-based analysis) produced during our time period of interest. For comparison, we also provide results from the motif-based analysis (Table 1). Of note, most birds did not sing 300 syllables in their first 20 motifs, thus the string-based analysis provided greater power for the conditional comparisons (Table 3).

**Figure 5:**
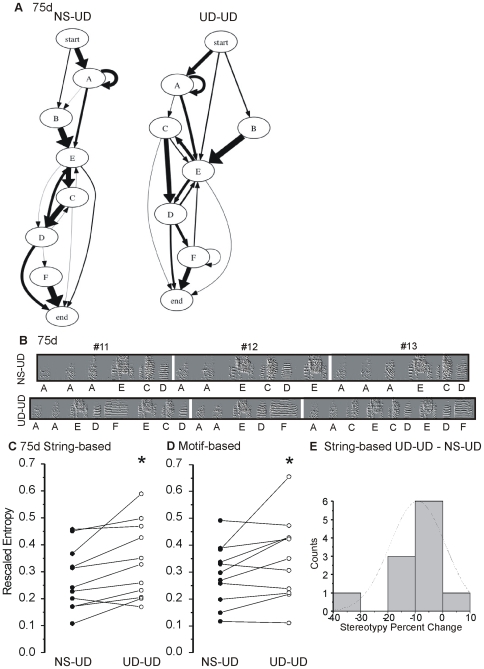
Sequence variability increases following continuous song practice at 75d. **A**) Markov chains for one bird in the two conditions illustrate the probability of syllable transitions observed using the motif-based analysis. Letters denote syllables. Line thickness corresponds to probability; thicker lines indicate greater probabilities. In the NS-UD condition, syllable E transitions to syllable C 83% (thick line) of the time whereas a thinner line represents a 16% probability that E ends the motif. By contrast, in the UD-UD condition, syllable E transitions to syllable C 50% of the time, to syllable D, 43%, and ends the motif 7%. In the NS-UD condition, syllable F occurs infrequently compared to the UD-UD condition. **B**) Examples of 3 consecutive motifs from the same 75d bird in the NS-UD and UD-UD conditions. Motifs occurred in the same chronological order in the selected 20 motifs analyzed (#11,12,13). Individual syllables are identified by letter. In the NS-UD condition, syllable A typically transitions to itself or to syllable E, and syllable C transitions most frequently to syllable D. By contrast, in the UD-UD condition, A also transitions to C (#13) as well. In the UD-UD condition, syllable F is observed (#11,12,13) and follows syllable D while in NS-UD, syllable D transitions to E (#12) or ends the motif (#11,13). **C–D**) Paired data scores for the NS-UD (filled circles) and UD-UD (open circles) conditions for each bird are represented by connected lines. At 75d, songs sung in the UD-UD condition exhibited greater variability (*, 2-tailed paired bootstrap ‘Rescaled Entropy’) compared to the NS-UD condition for both the string- (C, p<0.005) and motif-based analysis (D, p<0.05). **E**) Histogram reveals the percent change in stereotypy between the conditions.

At 75d, greater sequence variability was observed in the UD-UD condition. An example of these conditional differences is shown in Fig. 5 A which illustrates Markov chains produced from the NS-UD versus UD-UD conditions in one bird (Black288). Figure 5 B shows spectral derivatives of motifs from the same bird with the basic root sequence “AAAECD”. Songs sung in the UD-UD condition typically had longer motifs with additional repeated syllables and, as shown for this bird, frequent occurrences of unique syllables (Fig. 5 B, syllable F) rarely present in the NS-UD condition. Analysis of the 75d string- and motif-based data showed higher entropy and lower stereotypy scores in the UD-UD condition under standard and weighted measures (n = 11, standard string- p<0.005 and standard motif-based stereotypy, p<0.05; Fig. 5 C–D, Table 1; see Table S3 for weighted measures). At 75d, the distribution of percent changes is shifted towards negative values, reflecting increased sequence variability in the UD-UD condition (Fig. 5 E).

### Similar Trends at 65d Reflect Higher Overall Song Variability

Conditional differences in variability were also measured in these same birds at 65d. Similar to the 75d data, syllable analyses as well as the motif- and clip/string-based analyses showed a trend toward lower similarity, accuracy and stereotypy scores (8/11 measures) and higher entropy scores (2/11 measures) in the UD-UD condition (Table 1, Figure S2 B–D). However, statistical significance was only observed using the clip-based similarity score (n = 10 birds, p<0.05, Fig. 4 B). Since there were no overall differences in the syllable analyses, individual acoustic features are not reported.

The detection of statistically significant differences between conditions at 65d were likely precluded by the high within-condition variability of the data at this age (Fig. 4 C, Figs. S2, S3). The standard deviations (SDs) of song measures were higher at 65d relative to 75d when testing differences across the NS-UD and UD-UD conditions (65d vs. 75d SD for motif data: similarity: 4.4 vs. 3.2; accuracy: 2.0 vs. 0.8; stereotypy: 11.9 vs. 8.8). Critically, there was low power to detect conditional differences in 65d similarity, accuracy and stereotypy scores even if the number of birds is doubled prospectively (Table 3). With 20 birds per condition, the power only increases from 7%, 5%, and 11% to 32%, 37%, and 28%, respectively. By contrast, at 75d, the power to detect conditional differences in the present data is much higher (motif: 77%, 97%, and 52%, respectively; Table 3). Further evidence of high variability at 65d irrespective of condition came from the observed developmental shift in which song became more accurate and stereotyped between 65d and 75d (see below).

### Fine Tuning of Song Phonology over Development

Given the greater overall variability at 65d, we hypothesized that songs sung at 75d would exhibit higher phonology scores (less variability) than at 65d, reflecting progression of song development. Unexpectedly, phonological features of syllables did not differ significantly across the two ages in either condition although scores trended lower in the 65d data (n = 9, p>0.05, Table 4). For the motif- and clip-based analyses, as predicted, accuracy scores were higher at 75d than at 65d for the NS-UD condition (motif-based: NS-UD: p<0.005; clip-based: NS-UD: p<0.01) as well as for the motif-based analysis in the UD-UD condition (p<0.05). A strong trend was observed for the clip analysis (p = 0.052, Table 4). There was no difference in similarity scores although again a strong trend was evident in the NS-UD condition under the clip-based analysis (p = 0.052; Table 4). Power analysis revealed higher power in the NS-UD condition for detection of age differences (Table 3).

### Developmental Rise in Sequence Stereotypy from 65d to 75d

Syllable sequencing became more stereotyped with age in the NS-UD condition using standard motif- and string-based analyses (stereotypy, p<0.01) but not in the UD-UD condition (p>0.05; Table 4). However, when the weighted stereotypy measure was calculated, no age effect was observed in either the NS-UD or UD-UD condition (p>0.05, Table S3).

### Lack of Conditional Effect in Adults

The power to detect conditional differences in the 65d data was hindered by the high overall variability. In a separate group of adult birds, mature song exhibited low variability within each condition with higher similarity, accuracy, and stereotypy scores compared to juvenile birds. Even when the most stable songs sung by juvenile birds (i.e. at 75d under the NS-UD condition) were compared with those of adults, adult songs were more stable for similarity and stereotypy scores (p<0.01) with a trend for accuracy (p = 0.08). No differences were observed between the NS-UD and UD-UD conditions in these adults (p>0.05, Table 5). Power analysis of the motif-based adult data revealed low power (Table 5) for similarity (33.0%), accuracy (18.0%), entropy (5.6%), and stereotypy (4.7%) likely due to low or lacking between-condition variability. When the number of adults is prospectively tripled to 18, similarity is the only measure that yields acceptable power (90%; versus 30% for accuracy, 16% for both entropy and stereotypy).

## Discussion

Undirected song has been likened to vocal practice [19], [43], whereby variability enables trial and error learning and, ultimately, a desired motor output [44]–[46]. In zebra finches, expression levels of a number of known genes (Arc, Egr-1, Fos, SytIV), change during UD singing [18]–[26], [29], including the speech and language-related gene, FoxP2 [27], [28], (Teramitsu et al. companion article). This link between gene expression and mechanisms underlying song plasticity warrants investigation.

Here, we asked whether vocal practice under behaviorally-driven conditions, would increase vocal variability, at two ages during sensorimotor learning. In line with our prediction, we observed greater variability of song syllables in the UD-UD condition relative to the NS-UD condition at 75d, with similar trends at 65d. These results were observed in both the motif- and clip-based analyses, providing a high level of confidence in these findings and suggesting that 75d affords a developmental ‘sweet-spot’ for detection of vocal variability with practice.

We could not measure both behavior and FoxP2 levels simultaneously in the same bird, and did not specifically manipulate FoxP2 levels. However, our conditions for sampling song correspond to times of behavioral regulation of FoxP2 mRNA and protein [27], [28]. A strength of this approach is the relative lack of experimental intervention as we relied on behaviorally-induced changes in order to provide ethologically relevant results. But these changes inherently affect many molecules, including those noted above and previously shown to be regulated by song. Moreover, any effect of FoxP2, which is a transcription factor, raises the question of how its transcriptional targets relate to behavioral variability. Rather than operating independently, it is likely that networks of molecules are co-regulated by singing to integrate refinement versus reinforcement of vocalizations during song learning [47].

At 75d, syllable phonological scores were lower in the UD-UD condition and had a broader distribution than scores from the NS-UD condition, indicating more vocal variability (Fig. 2). Upon further inspection of the data, we found that the conditional differences were driven mostly by increased variability in pitch, pitch goodness, Wiener entropy, and amplitude. Our analysis included both ‘noisy’ and ‘flat harmonic’ syllables; removal of the flat harmonic syllables from the calculation still yielded significance, suggesting a developmental sculpting of syllables with high entropy. Haesler and colleagues (2007) [35] showed that the syllables of birds with constitutive *FoxP2* knockdown of ∼20% of area X neurons via RNA interference, were less similar to their tutors', and differed in duration and Weiner entropy from control birds. Here, we show modulation of individual syllable features on a more rapid (i.e. 2 hour) time scale, under conditions previously shown to naturally lower area X FoxP2 levels (Teramitsu et al., companion article). Thus, FoxP2 function in area X is potentially part of the mechanism underlying the conditional modulation of phonology observed here. Within area X, FoxP2 is expressed in medium spiny neurons [30], [48]. One working hypothesis is that lowered FoxP2 levels in these neurons during UD singing promote their excitability. Area X is part of the anterior forebrain pathway, whose output is to premotor nucleus RA. In this pathway, area X medium spiny neurons mediate GABAergic inhibition of projection neurons *in vitro* [49], [50] and *in vivo* during UD song [51]. These neurons, in turn, tonically inhibit DLM in both juveniles [52] and adults [53], [54]. Disinhibition of DLM could drive excitation of LMAN and subsequently, premotor nucleus RA [55]. In turn, these variations in pre-motor activity in RA would regulate variations in spectral structure [46].

The variability discussed here is on a time-scale of hours rather than the minute-to-minute changes that also occur [39], [40]. Perhaps neuromodulators that promote the more rapid changes simultaneously set in motion slower processes such as altered gene regulation. Neuromodulatory candidates include dopamine [56]–[58] and norepinephrine [59] as well as signaling cascades initiated by glutamatergic input from song control nucleus HVC [38], [60]–[62]. The integration of such neuromodulatory influences over time could result in downstream transcriptional changes that include FoxP2, Egr-1 [27], [18], [19], and other genes that promote neuroanatomical and behavioral changes potentially underlying song learning.

Continuous vocal practice in the morning also increased sequential variability at 75d as revealed by both the motif- and string-based analyses. For a given syllable in the UD-UD condition, it was more difficult to predict what the next syllable would be and, in some cases, there were more possible transitions than in the NS-UD condition (e.g., Fig. 5 A). Over a greater developmental timecourse, morning peaks of UD song production and the high amount of singing by juveniles have been reported to reduce sequencing errors in adulthood [63]. Developmental improvement in sequencing (between 65 and 75d) was also obtained here (see below). Haesler and colleagues also used a string-based method to analyze sequencing, but did not report increased syllable sequence variability in the songs of FoxP2 knock-down birds compared to controls [35]. The different outcomes between studies likely reflect methodological differences, potentially including that we did not specifically manipulate FoxP2.

Detection of significant conditional differences was limited to data obtained at 75d because of the high within-condition variability in the 65d data. Data collected at 65d did show trends for greater variability in the UD-UD condition, with significant differences in similarity revealed by the clip-based-analysis. The improved detection of conditional differences using the one second clips at 65d was surprising given that this analysis appeared to provide a stricter test of conditional differences for the 75d data, reflected in higher p-values relative to the motif-based analysis. Hence, we did not expect the clip-based analysis at 65d to be more sensitive to any conditional difference in phonology. This unexpected finding suggests that at 65d, there is a gross level of phonological variability that can only be detected in SAP by disregarding motif structure. Further, these comparisons suggest that fine-grained phonological tuning, reflected by accuracy scores, occurred less at 65d when compared to 75d, while coarser tuning, reflected in similarity scores, occurred at roughly the same level at both ages. We expand on these interpretations in Appendix S1.

Power analysis revealed low power for detection of conditional differences at 65d even when prospectively increasing the number of birds per condition, likely due to high within-condition variability obscuring any between-condition effect. The adult data, like the 65d data, also showed low power for detection of conditional differences and increasing the number of adult birds per condition does not substantially increase the power. Unlike in the 65d data, however, in adults, it was the low between-condition difference that diminished the power. We note that lack of robust conditional differences at 65d and in mature song rules out that increased variability at 75d in the UD-UD condition reflects singing fatigue. Moreover, birds sang similar amounts immediately after 2 hours in both conditions, also arguing against any fatigue. While we did not observe conditional effects on song variability in adult birds, other labs have documented rapid effects of social context on the variability in fundamental frequency (FF) [39]. Here, we did not examine social context and were unable to make meaningful comparisons of the adult FF due to low sample size for the flat harmonic syllables.

The lack of a conditional effect in adults is surprising, given the equivalent amount of singing-induced FoxP2 down-regulation in juveniles (Teramitsu et al., companion article) and adults [27], [28]. It could be that FoxP2 is only involved in behavioral variability during song learning, or that its involvement in adult variability is too subtle to detect with our current methods, or that FoxP2 has nothing to do with behavioral variability. The FoxP2 knock-down study in birds [35], coupled with the 75d and, to a lesser extent, the 65d data shown here support some role for FoxP2 in song variability. Given that the correlation between amount of singing and FoxP2 down-regulation is stronger in juveniles than in adults (Teramitsu et al. companion article), it may be that any FoxP2 effects are more profound during sensorimotor learning, which relies to a greater extent upon variable motor output than the maintenance of adult song. Likewise, the effects of deafening or of LMAN lesions are easier to detect in young birds, although a similar role (i.e. hearing for learning/maintenance; LMAN for variability) is posited at each timepoint [64]. Potentially, after song crystallization there is a relative de-coupling of singing and gene network activity important for variability during song development. In this light, it would be interesting to compare the molecular networks that are activated during undirected singing in juvenile versus adult area X, and in birds in which FoxP2 levels have been genetically manipulated.

A comparison of the 65d versus 75d data in the same group of birds revealed age-related increases in song stability, as expected for juveniles undergoing sensorimotor learning. For within-condition comparisons, both the motif- and clip-based analyses showed higher accuracy scores at 75d compared to 65d. This is consistent with the detection of conditional differences in the 75d data. Unexpectedly, at the syllable level, phonological scores were not significantly higher at 75d compared to 65d. The developmental increase in accuracy scores observed at the motif/clip-based level, but not at the syllable level, may reflect a more comprehensive coarse tuning of all syllables versus fine-tuning of select syllables over the ten day time period. Using the standard measure, sequence stereotypy also increased from 65d to 75d in both conditions using motif- and clip-based analyses. In contrast, no developmental improvement in sequencing was observed using the frequency weighted measure (Table S3), which effectively de-emphasizes infrequently occurring syllables (see Methods). This suggests that one source of increased sequence stereotypy in 75d songs is a decrease in the number of infrequently sung syllables.

The maturational increase in stability observed here is consistent with other studies (c.f. [40], [63]). As previously noted, Derégnaucourt and colleagues (2005) found that the magnitude of phonological deterioration during a night of sleep diminishes with age and that juveniles with the greatest overall morning deterioration ultimately produced the best imitations of their tutor's songs [12]. Processes that promote this deterioration may include song replay during sleep, as noted by those authors. Thus, multiple forms of ‘practice’ (either during sleep, or during the first two hours of singing in the morning) may promote variability. One experiment conducted in the Derégnaucourt study closely matches the behavioral conditions used here. Birds on one day were prevented from singing for 2 hours in the morning and then the percent of vocal changes was compared to songs sung the day previous or the day after, when birds were allowed to sing continuously. Although there was no evidence for a conditional effect on that measure of variability, we note that the age of the birds tested was 50–57d. As our power analysis demonstrates, we would be unable to detect conditional differences at this age using the number of birds (n = 6) employed in that experiment. Thus, as with morning deterioration, the variability observed following morning song practice may ultimately enable more precise emulation of the tutor song, although we did not test this. If so, then according to the FoxP2 ‘plasticity gate’ hypothesis, we would predict a resurgence in FoxP2 levels with continued vocal practice beyond two hours, to enable stabilization of optimal motor patterns. The idea that this type of experience-dependent trial and error learning ultimately leads to improved motor performance has been postulated previously [65].

Moving forward, one should not consider single genes in isolation, but in the context of other genes and gene networks in humans [66], [67] and in songbirds [47], [26]. During brain formation, FoxP2 transcriptional targets, such as contactin-associated protein like-2 (CNTNAP2) [68] are emerging that appear vital to the neural patterning required for human language [69], [70] and may also be important for song circuitry [71]. During learning, it is likely that birdsong and speech also share common molecular mechanisms. Vocal motor variability and exploration on multiple time scales likely facilitates the refinement of vocal output during development, and the maintenance of previously learned, ultra-precise, motor patterns throughout life. Indeed, motor memory stabilization in adult humans relies upon intermittent practice [72], as in songbirds [44]. An important starting point will be to uncover gene targets or ensembles vital for motor variability in general, and more specifically, phonological and sequence variability related to learned vocalizations.

## Supporting Information

Appendix S1 Methods and results.
(0.03 MB DOC)

Table S1 Motif-based scores and test statistics for all statistical methods. Means are reported along with exact p-values from Student's paired t-test (parametric) and Wilcoxon signed-rank and bootstrap statistics (nonparametric) for 2-tailed tests. Significant p-values are highlighted in bold face type.
(0.09 MB DOC)

Table S2 Unweighted clip- and string-based scores and test statistics. Means are reported with exact p-values from Student's paired t-test (parametric), and Wilcoxon signed-rank and bootstrap statistics (nonparametric) for 2-tailed tests. Significant p-values are highlighted in bold face type.
(0.08 MB DOC)

Table S3 Frequency-weighted clip- and string-based scores. Means are reported with exact p-values from Student's paired t-test (parametric) and Wilcoxon signed-rank and bootstrap statistics (nonparametric) for 2-tailed tests. Significant p-values are highlighted in bold face type.
(0.09 MB DOC)

Figure S1 Subset of phonological features that did not differ between conditions at 75d. Box plots show the mean scores (middle of the box), standard error (top and bottom of the box), and upper and lower 95% confidence intervals (whiskers). Data scores for the NS-UD (filled circles) and UD-UD (open circles) conditions for ∼3 syllables from each bird (30 syllables total) are represented by individual points. Mean CV scores were obtained from 25 renditions of the same syllable. No differences in CV (p>0.05) were observed for syllable mean frequency, duration, or frequency modulation (FM).
(0.62 MB TIF)

Figure S2 Syllable scores did not differ between conditions at 65d. A) Paired data shows similarity scores for the NS-UD (filled circles) and UD-UD (open circles) conditions for each bird at 65d. Individual points represent a mean syllable score from a single bird. Although the mean values in the UD-UD condition were lower than NS-UD means, the differences were not significant (2-tailed paired bootstrap, p>0.05). B–D) Histograms show the distribution of phonological scores for all 25 syllables from 10 birds (2-tailed paired bootstrap, p>0.05). For both conditions, scores were broadly distributed, reflecting greater overall variability in song at 65d relative to 75d.
(1.06 MB TIF)

Figure S3 No conditional differences were observed in motif and sequence variability at 65d. A) Motif similarity and accuracy scores for 65d were similar between the NS-UD and UD-UD conditions (2-tailed paired bootstrap, p>0.05). B–C) Entropy scores for the string- and motif-based analysis were similar between the two conditions (2-tailed bootstrap, p>0.05). D) Histogram depicts the percent change in the string-based scores, showing bi-directional distribution.
(0.85 MB TIF)
