# Supplementary material for: Song Practice Promotes Acute Vocal Variability at a Key Stage of Sensorimotor Learning
Source: PLoS One. 2010 Jan 6;5(1):e8592. doi: 10.1371/journal.pone.0008592 (PMC2797613; doi:10.1371/journal.pone.0008592)
Supplement: Appendix S1 — Methods and results. (0.03 MB DOC) [file pone.0008592.s001.doc]

**Appendix S1, Methods**

*Phonology: Comparison of motif-based vs. clip-based analyses*

SAP was originally written to track the learning of a young zebra finch's song during development by comparing it to the song of his tutor [1]. As such, asymmetric similarity scoring in SAP assumes one sample is the "template", and searches for sections of the template in the "pupil" sample, i.e. SAP searches for regions of the pupil song that are acoustically similar, above a predefined significance threshold, to regions of the tutor song. A score is assigned based on how much of the "template" data can be found in the "pupil" sample. By comparing a list of song samples to itself, as we do here, each sample is both 1) considered the "template", serving as the baseline to which all the other "pupil" samples are compared, and 2) considered a "pupil" sample during every other sample's turn as the "template." Since the same list of files is being compared to itself, higher average scores are interpreted as lower acoustic variability for the time period during which those song samples were recorded, while lower average scores are interpreted as higher variability.

Controlling for the length of song samples is important since the degree of similarity between two song clips is partly a function of the length of the clips. For example, in a bird with two versions of his motif, one long and one short, with equal acoustic variability, each occurring roughly 50% of the time, any collection of 20 motif renditions will contain some short and some long motifs. Comparing a list of 20 motifs to itself will produce at least two populations of scores, one being long-long and short-short comparisons (these will tend to yield higher similarity scores), the other being short-long and long-short comparisons (these tend to yield lower scores). Also, since SAP assumes one sample is the "template" and one the "pupil", if a short motif is the template, SAP is more likely to return a higher score than if a long motif is the template. This can lead to the short-long/long-short score distribution having a wider spread than the long-long/short-short distribution. It could also mean that there are really three score distributions, long-long/short-short, long-short, and short-long, or more depending on the variability of motif length within the long and short sample populations. Any of the above scenarios is problematic for statistical analysis if one desires only one representative number for a given batch run on a given bird. If there are really two or more distributions of scores it is unclear if the mean is a good descriptor of the data. Thus, performing the one second clip-based analysis is a reliable alternative. One second was chosen since it is roughly the average length of a zebra finch motif. The number of 30 clips was arrived at empirically; first running 10x10, 15x15, 20x20, etc until the mean score saturated.

*Sequence comparison*

In the motif analysis, syllable transition probabilities were computed on a different number of syllables for each bird, even though the number of motifs was the same. End transitions were included in the calculations. Since few of the birds sang 300 syllables over the course of their first 20 motifs, the string-based analysis increased the number of syllables used in the calculations and kept the number constant without inclusion of motif end transitions.

MATLAB code: http://www.physci.ucla.edu/research/white/links.html

**Appendix S1, Results**

***65d similarity score lower in the UD-UD condition using the clip-based analysis***

In our analysis of phonological variability, we found that similarity and accuracy scores were reduced at 75d in the UD-UD condition, indicating more variability after two hours of continuous vocal practice. At 65d, trends in the data indicated lower scores in the UD-UD condition but only the clip analysis revealed a statistically significant drop in similarity.

Some aspect of the motif-based analysis masked the decrease in 65d UD-UD similarity scores seen in the clip-based analysis. The large variability in motif length at 65d that led to binomial score distributions within birds was more apparent in similarity than in accuracy scores, perhaps reflecting the fact that similarity scores are derived from larger sampling windows than are accuracy scores. If this is the case, similarity scores would be highly sensitive to fluctuations in sample length, and perhaps the larger 65d motif length variability, coupled with greater general phonological variability at 65d, resulted in the obfuscation of a subtle 65d UD-UD increase in gross phonological variability. Also, while the clip-based analysis controlled for sample length, it ignored the inherent motif structure of song. In fact, this was the very intent of performing this analysis. Even though the motif-based song samples varied in length, each sample contained approximately the same group of syllables in essentially the same order, usually beginning and ending with the same syllables. The contents of the one second clips were more arbitrary, composed of any number of syllables occurring in that one second. While there was certainly still some patterning with regard to syllable identity and number in the clips, the acoustic heterogeneity between samples was almost certainly greater than in the motif samples. The degree of increased heterogeneity for each bird would have been a function of motif length and number of unique syllables. This heterogeneity could be seen as indicative of a less biased sampling procedure, and may explain why the clip-based analysis was more sensitive to coarse changes than the motif-based analysis at 65d. Both detected subtle statistically-significant changes later, at 75d, speaking to a more obvious increase in fine grained phonological variability in general, and to overall increased relative variability in the UD-UD condition at this age.

**Reference**

1. Tchernichovski O, Nottebohm F, Ho CE, Pesaran B, Mitra PP (2000) A procedure for an automated measurement of song similarity. Animal Behaviour 59:1167-1176.
