# Supplementary material for: Song Practice Promotes Acute Vocal Variability at a Key Stage of Sensorimotor Learning
Source: PLoS One. 2010 Jan 6;5(1):e8592. doi: 10.1371/journal.pone.0008592 (PMC2797613; doi:10.1371/journal.pone.0008592)
Supplement: Table S1 — Motif-based scores and test statistics for all statistical methods. Means are reported along with exact p-values from Student's paired t-test (parametric) and Wilcoxon signed-rank and bootstrap statistics (nonparametric) for 2-tailed tests. Significant p-values are highlighted in bold face type. (0.09 MB DOC) [file pone.0008592.s002.doc]

**Table S1.** **Motif-based scores and test statistics for all statistical methods.**
