# Supplementary material for: Song Practice Promotes Acute Vocal Variability at a Key Stage of Sensorimotor Learning
Source: PLoS One. 2010 Jan 6;5(1):e8592. doi: 10.1371/journal.pone.0008592 (PMC2797613; doi:10.1371/journal.pone.0008592)
Supplement: Table S2 — Unweighted clip- and string-based scores and test statistics. Means are reported with exact p-values from Student's paired t-test (parametric), and Wilcoxon signed-rank and bootstrap statistics (nonparametric) for 2-tailed tests. Significant p-values are highlighted in bold face type. (0.08 MB DOC) [file pone.0008592.s003.doc]

**Table S2.** **Unweighted clip- and string-based scores and test statistics.**
