# Supplementary figures and images for: Song Practice Promotes Acute Vocal Variability at a Key Stage of Sensorimotor Learning
Source: PLoS One. 2010 Jan 6;5(1):e8592. doi: 10.1371/journal.pone.0008592 (PMC2797613; doi:10.1371/journal.pone.0008592)

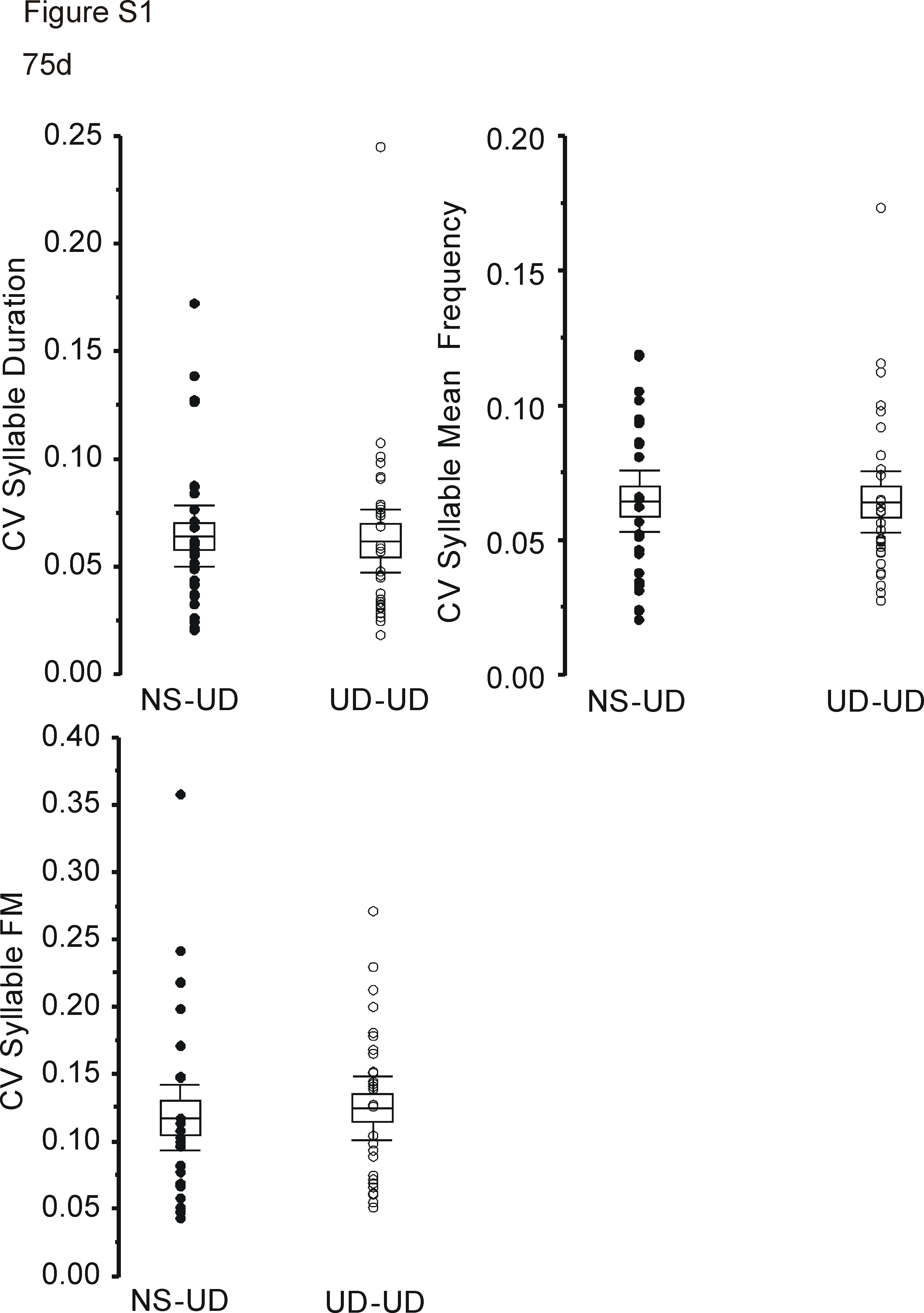

Supplement: Figure S1 — Subset of phonological features that did not differ between conditions at 75d. Box plots show the mean scores (middle of the box), standard error (top and bottom of the box), and upper and lower 95% confidence intervals (whiskers). Data scores for the NS-UD (filled circles) and UD-UD (open circles) conditions for ∼3 syllables from each bird (30 syllables total) are represented by individual points. Mean CV scores were obtained from 25 renditions of the same syllable. No differences in CV (p>0.05) were observed for syllable mean frequency, duration, or frequency modulation (FM). (0.62 MB TIF) [file pone.0008592.s005.tif]

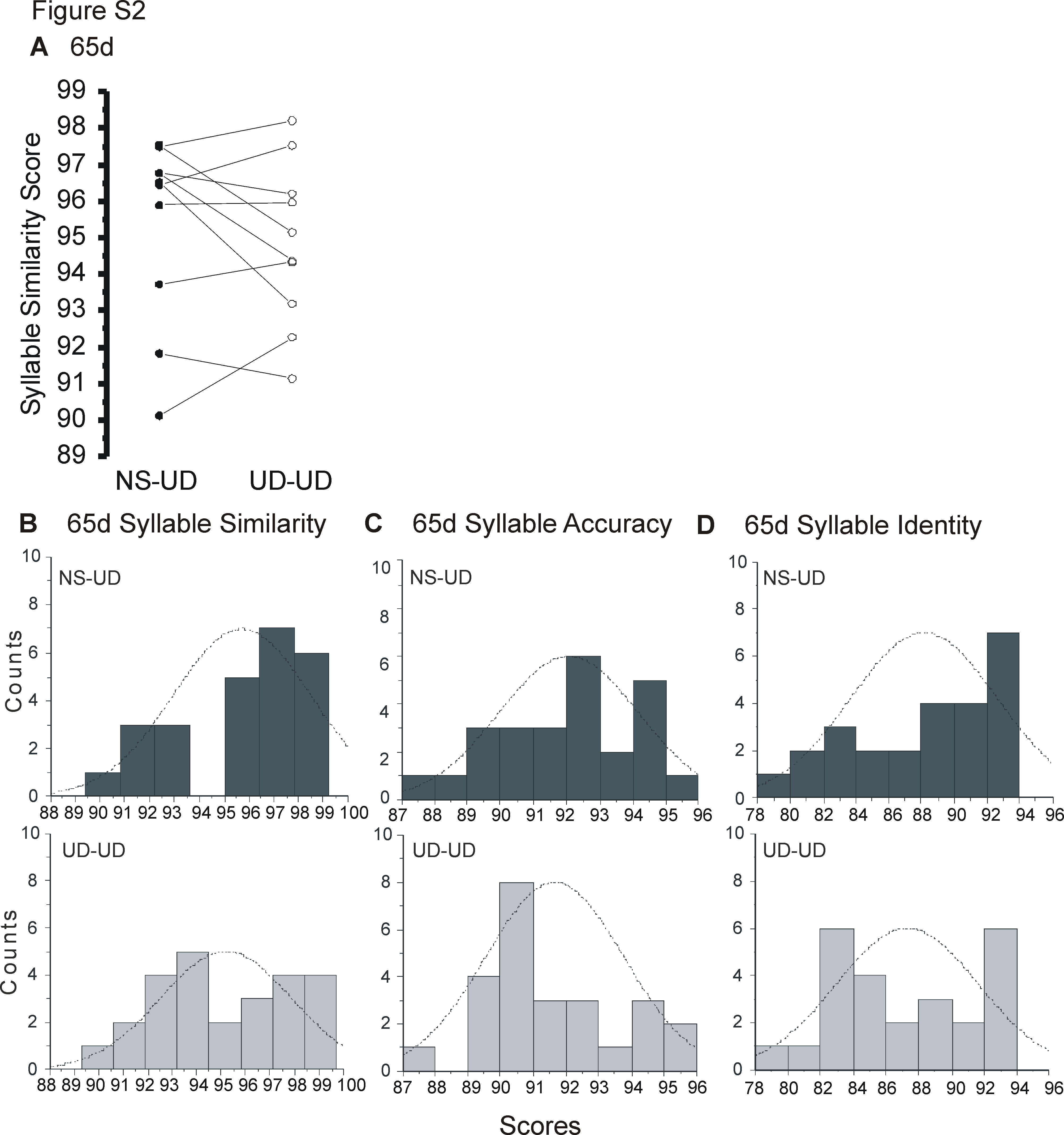

Supplement: Figure S2 — Syllable scores did not differ between conditions at 65d. A) Paired data shows similarity scores for the NS-UD (filled circles) and UD-UD (open circles) conditions for each bird at 65d. Individual points represent a mean syllable score from a single bird. Although the mean values in the UD-UD condition were lower than NS-UD means, the differences were not significant (2-tailed paired bootstrap, p>0.05). B–D) Histograms show the distribution of phonological scores for all 25 syllables from 10 birds (2-tailed paired bootstrap, p>0.05). For both conditions, scores were broadly distributed, reflecting greater overall variability in song at 65d relative to 75d. (1.06 MB TIF) [file pone.0008592.s006.tif]

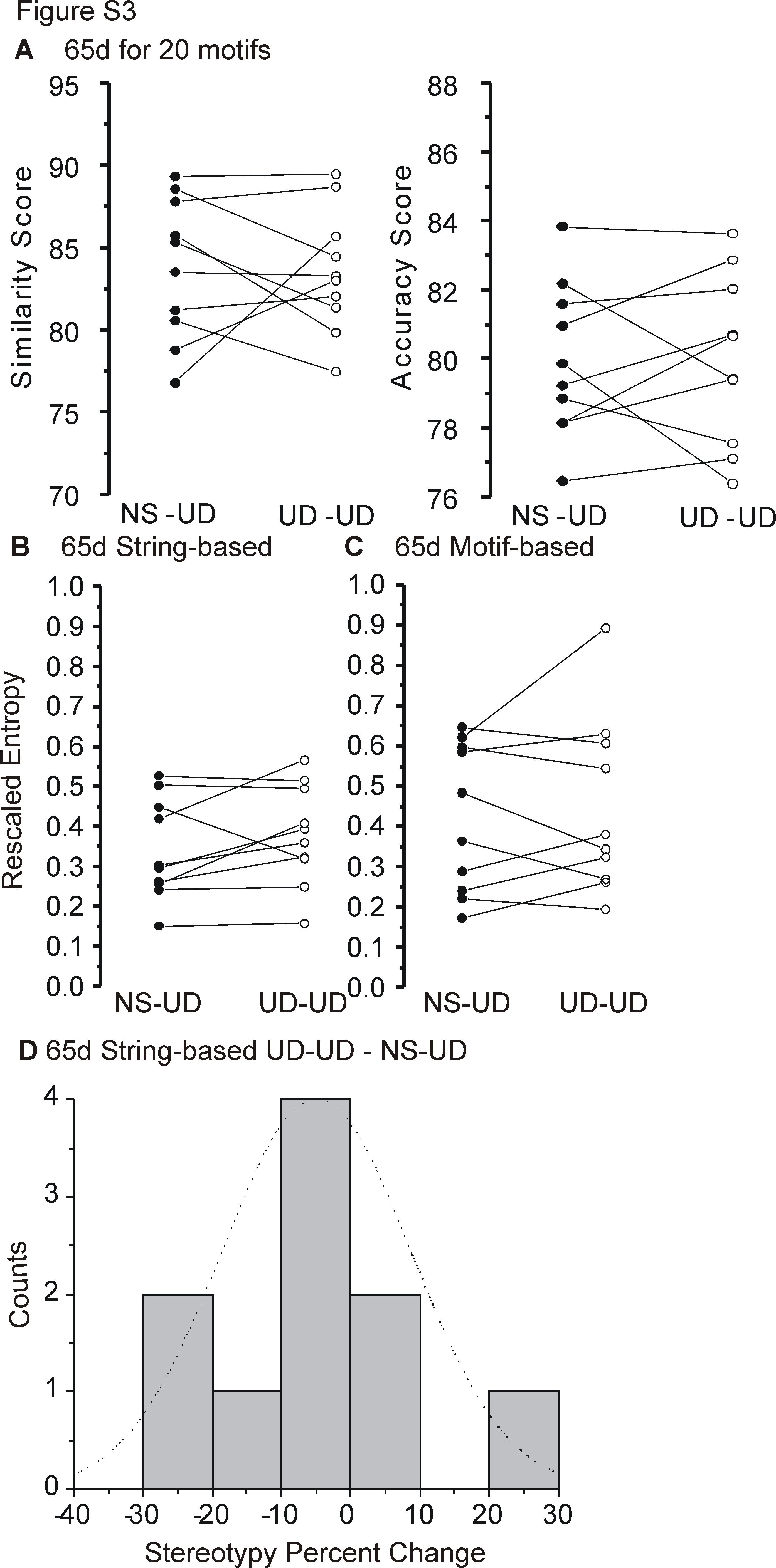

Supplement: Figure S3 — No conditional differences were observed in motif and sequence variability at 65d. A) Motif similarity and accuracy scores for 65d were similar between the NS-UD and UD-UD conditions (2-tailed paired bootstrap, p>0.05). B–C) Entropy scores for the string- and motif-based analysis were similar between the two conditions (2-tailed bootstrap, p>0.05). D) Histogram depicts the percent change in the string-based scores, showing bi-directional distribution. (0.85 MB TIF) [file pone.0008592.s007.tif]
